# Construction and analysis of a leaf cDNA library from cold stressed rubber tree clones

**DOI:** 10.1186/1753-6561-5-S7-P24

**Published:** 2011-09-13

**Authors:** Carla Da Silva, Tatiana Campos, Erivaldo Scaloppi, Mírian Maluf, Paulo Gonçalves, Anete Souza

**Affiliations:** 1 Molecular Genetics and Analysis Laboratory, Genetics and Evolution Department, Biology Institute, CBMEG/UNICAMP, Brazil; 2 Agroforestry Research Center from Acre, Embrapa Acre, Brazil; 3 São Paulo Northwest Regional Research Center, APTA, Brazil; 4 Alcides Carvalho Coffee Center - IAC/Embrapa, Brazil; 5 Rubber Tree Breeding Program – IAC/Embrapa, Brazil; 6 Plant Biology Department, Biology Institute, UNICAMP, Brazil

## Background

Rubber tree [*Hevea Braziliensis* (Willd. ex Adr. de Juss.) Muell-Arg.], a native species of the Amazon Rainforest in South America, belonging to the Euphorbiaceae family, is world’s major source of natural latex. Brazil, despite being the center of origin and diversity of the species and latex main producer at the end of XIX century, nowadays imports around 70% of the rubber consumed in the country. Although the Amazonian basin provides optimal conditions for rubber plantation, the occurrence of South American leaf blight (SALB) disease, caused by the ascomycete *Microcyclus ulei*, limits the rubber production in this region. Therefore, rubber plantation has been extended to suboptimal areas that are prominently located in northeast India, Vietnam, southern China and southern plateau of Brazil. Besides the new conditions for crop development, these new areas present stress conditions like low temperatures, dry periods and wind. It was shown that low temperatures affect the development and latex production of rubber trees. Breeding programs have been searching for clones adapted to these suboptimal areas. A complete cycle of breeding and selection takes at least 20 years to succeed in obtaining a superior clone. As such, development of new methods for early evaluation is essential to reduce and optimize the breeding management. Aiming to help in the development of these methods, in this work, we have constructed a leaf cDNA library to study ESTs (Expressed Sequence Tags) from cold stressed rubber trees. The originated sequences from this library can be used to create an ESTs databank and will provide genetic knowledge about how rubber trees cope with chilling stress, being also a valuable source of polymorphic molecular markers, such as SSRs and SNPs. Some of these markers may be associated to chilling tolerance, and early evaluation would be possible in young rubber tree clones.

## Methods

The young *Hevea Braziliensis* Muell. Arg. clones GT1, IAN873, PR255 and PB217 were obtained from Agronomic Institute of Campinas (IAC). GT1, PR255 and PB217 clones are genitors of mapping populations of ongoing projects at Molecular Genetics and Analysis Laboratory and IAN873 clone showed chilling tolerance in field. The 24h cold treatment was peformed in a diurnal growth chamber (8°C, 12h photoperiod). The collected leaves were previously wrapped up in tinfoil to prevent transcript redundancy. Leaves were sampled in intervals of 6h, 10h and 24h and total RNA was extracted by LiCl precipitation. Total RNA extracted from clones were pooled in equal amounts, according to time of sampling. The In-fusion SMARTer cDNA Library Construction Kit (Clontech) was used to construct the cDNA library. A sequenced plaque (96 clones) of each sub-library (6h, 10h and 24h) was chosen for a preliminary analysis of the library. Sequences were analyzed and clustered by the DNAStar package (DNAStar, Inc.) and Blast2Go software (http://www.blast2go.org) was used to annotate the contigs and singlets. The resulting ESTs were screened for microsatellite regions using Simple Sequence Repeat Identification Tool (SSRIT) (http://www.gramene.org/db/markers/ssrtool).

## Results and discussion

**Figure 1:**
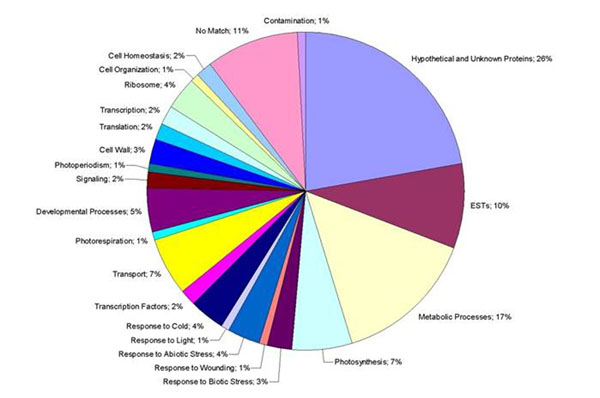
Relative distribution of annotated ESTs into functional categories

The cold stressed leaf cDNA library has around 6,000 clones and is sub-divided into three libraries: “cold-6h”, “cold-10h” and “cold-24h”, having 2,000 clones each. The average fragment lengths from these libraries are 780bp, 625bp and 930bp, respectively. In order to perform a preliminary analysis of the libraries expression profiles, 96 sequenced clones from each library were chosen randomly. Among the 288 sequences analyzed, 87 were discarded due to sequencing reaction failure or sequencing slippage. Clustering of the remaining 201 sequences generated eight contigs and 180 singlets. All contigs and singlets were annotated and distributed into functional categories. “Hypothetical and Unknown Proteins” was the major functional category, containing 52 sequences (26%). Most of these sequences (25) belong to “cold-24h” library. Second larger functional category was “Metabolic Processes”, constituted by 34 sequences (17%). It was also in that category that libraries showed a visible difference among them: while about 20% of the sequences of libraries “cold-6h” and “cold-10h” is involved in cellular metabolic processes, in the “cold-24h” library this number falls to 13%. Chilling stress mainly causes alterations in metabolic processes and decrease in enzymatic activities. An exposure of 24 hours to low temperatures seems to be enough to trigger such a change in stressed rubber trees, which maybe could explain the higher number of hypothetical proteins in that library; however analysis of more sequences is necessary to confirm the results. Sequences that presented no similarity to any sequence in GenBank database were grouped in the “No Match” category, which is third in number of sequences (22; 11%). These sequences may be specific to *Hevea Braziliensis* and several presented at least one ORF that could be translated. The relative distribution of all annotated ESTs into functional categories can be seen in figure 1. The presence of microsatellite regions in the sequences was also verified and 30 ESTs sequences bearing SSR regions (15%) were found: 13 dinucleotide (43%), 9 trinucleotide (30%) and 8 tetranucleotide motifs (27%). Microsatellite regions were present mostly in hypothetical protein sequences (13; 43%) and all of them were in the UTR regions. Primers for these sequences are being designed in order to evaluate their polymorphism. The sequencing process of the cold stressed leaf cDNA library is still ongoing. The comparison of a small number of sequences showed a difference in expression pattern between the sub-libraries that may be confirmed by a wide analysis of their sequences. A preliminary analysis for microsatellite regions suggested that around 15% of the sequences may have potential polymorphic SSR markers. Moreover the ESTs generated will be a rich source for polymorphic SNP markers. SNP and SSR molecular markers will be developed from these data and will be used in *H. Braziliensis* genetic mapping. These markers could provide tools for early evaluation of cold tolerant rubber tree clones, among other genetic studies.

